# NOV story: the way to CCN3

**DOI:** 10.1186/1478-811X-4-3

**Published:** 2006-02-20

**Authors:** Bernard Perbal

**Affiliations:** 1 Laboratoire d'Oncologie Virale et Moléculaire, Case 7048, UFR de Biochimie, Université Paris 7 – D. Diderot, 2 place Jussieu, 75005 Paris-France

## Abstract

The principal aim of this historical review- the first in a new series- is to present the basic concepts that led to the discovery of NOV and to show how our ideas evolved regarding the role and functions of this new class of proteins. It should prove particularly useful to the new comers and to students who are engaged in this exciting field. It is also a good opportunity to acknowledge the input of those who participated in the development of this scientific endeavour

## Introduction

In this manuscript I am presenting milestones in our discovery of NOV (CCN3), a founding member the CCN family of regulatory proteins [1], now known to be composed of six members that play critical roles in normal fundamental biological processes including angiogenesis, wound repair, regulation of cell spreading proliferation and survival [2,3]. Alterations in the expression of CCN genes are also associated with cancerogenesis [4,5].

### The past

In my opinion, the « nov story » finds its roots in 1982 with the molecular cloning of a MAV-1(N) (myeloblastosis associated virus type 1) proviral genome that was shown to specifically induce nephroblastomas when injected into day-old chickens [6,7]. As a fellow on leave from the CNRS at UCLA in the laboratory of Pr. M. Baluda, I had cloned both the v-myb oncogene [8] and c-myb proto-oncogene [9] and I became interested in the molecular basis for MAV-induced nephroblastomas, which resemble the Wilms' tumors [10]. The MAV strains that were used at this time were inducing nephroblastomas, lymphoid leukosis and osteopetrosis [11]. Because these strains were at best, plaque purified, it was not easy to assess the biological properties of MAV.

Back at that time, the idea prevailed that retroviruses induced tumors by inserting in the vicinity of cellular proto-oncogenes. Pioneer work of Hayward, Astrin and collaborators had opened the road for my interest in identifying the integration sites of MAV.

After my return to France, M. Brisac who was a student in my laboratory was given the task to characterize the MAV junction fragments in tumor DNA, with the help of Dr. G. Dambrine at the INRA who provided animal facilities and expertise in chicken pathology. Several tumors were obtained and analyzed both at the histological level by Dr. G. Plassiart and Pr. M. Wyers at the Ecole Vétérinaire de Nantes, and at the molecular level in my laboratory. Even though I had previously cloned a U3-specific probe for MAV, these studies were seriously complicated by the presence in the avian genome of endogenous sequences that were cross-reacting with the structural MAV probes that we used. Another student, J. Soret, who was working on v-myb in my group took over this study when M. Brisac left. He soon established that MAV nephroblastomas were polyclonal tumors and contained several rearranged proviral genomes [7].

The very high efficiency of nephroblastomas induction by MAV,100 % in 8 weeks, made it a unique model in which to study the molecular basis of its tumorigenic potential and to identify new potential oncogenes or tumor suppressors.

### The near past

At that time, V. Joliot, who came on as a graduate student in my laboratory, was given a project aimed at identifying the cellular and viral determinants for the restricted pathogenic potential of MAV-1(N). Part of her project included the cloning of cellular DNA fragments containing MAV proviral sequences, so-called junction fragments, in order to establish whether the expression of neighbouring genes would be activated or repressed upon MAV integration.

The classical strategy at that time, consisted in preparing genomic libraires of tumor DNA. A critical step in the procedure consisted in the preparation of good quality high molecular weight DNA from tumor tissues samples which are known to contain a significant amount of degraded material if not collected and kept under proper conditions [12]. A key point to our success in this approach was provided by the selection, as starting material, of three different tumors showing different developmental stages. I must say that the expertise of G. Dambrine, G. Plassiart and M. Wyers was pivotal in making this choice.

When a senior scientist, Dr. C. Martinerie, who previously worked on c-myb in my laboratory returned from a post doctoral stay in USA, I offered her to join the MAV project that V. Joliot had some difficulties to get moving. Eventually, the three libraires were prepared and their screening permitted the cloning of MAV-containing tumor DNA fragments. The next step in this strategy consisted in using these DNA fragments as probes on Northern blots of RNA species purified from avian normal and tumor samples. Only one junction fragment detected sequences that were differentially expressed in tumor and normal cells. In tumor 725 this probe detected high levels of a 2.0 kb RNA species that was not detected in normal adult kidney tissues from 8 week-old chicken [13].

A cDNA library of chicken DNA was screened by V. Joliot and C. Martinerie to isolate a cDNA species corresponding to the sequences that were highly expressed in nephroblastoma 725. When this cDNA was used as a probe, it was found that the corresponding sequences were highly expressed in all MAV-induced nephroblastomas [13]. Upon the suggestion of V. Joliot, the gene was designated « nov » for « nephroblastoma overexpressed ». The nov cDNA was encoded by a gene consisting of 5 exons which showed a high similarity with two other genes – chicken cef10 and the murine fisp12, subsequently  renamed CCN1 and CCN2 (see below) – previously reported to be immediate early genes whose expression was increased upon transformation of fibroblasts by the src oncogene or following serum stimulation of starved cells [14,15]. The murine ortholog of CEF10 had been cloned as a gene encoding a cystein-rich protein designated cyr61 [16] and the human ortholog of Fisp12 had been cloned as an immediate-early gene encoding a protein that was isolated from Huvec (human umbilical vein endothelial cells), on the basis of its cross reactivity with anti PDGF antibodies [17].

Since nov was disrupted by MAV in only one nephroblastoma, we hypothesized that the integration of proviral LTR sequences in the vicinity of nov might account for its overexpression in the other avian tumors. However, recent studies [18] have challenged this view and questioned the significance of nov overexpression in chicken nephroblastomas (see below).

Isabelle Joubert who joined my team to prepare a PhD, studied several other nephroblastomas induced by MAV 1(N) or by a strain of MAV2(O) that had been cloned by V. Joliot [19] and reached the conclusion that all tumors expressed high levels of nov. The coding frame of the full length nov mRNA species was not altered in the tumors that I. Joubert studied, therefore suggesting that high levels of a normal nov protein are expressed in nephroblastomas [10].

In the second part of her thesis project, I. Joubert attempted to establish whether MAV sequences were integrated in the vicinity of nov in tumors expressing high levels of full length nov mRNAs. Since our previous studies permitted analysis of only 20 kb of sequences surrounding MAV LTRs in tumor DNA, we decided to construct BAC (bacterial artificial chromosomes) libraries of tumor DNA. Unfortunately, this approach was unsuccessful and the question was left open until C.L. Li, an Assistant Professor from China, joined our laboratory (see below).

Since MAV-induced nephroblastomas constituted a unique model of the Wilms' tumor, a pediatric kidney tumor arising in 1:6000 birth [20], we became interested in establishing whether the expression of the nov gene was also elevated in these tumors.

As a first step along this line, I had established a collaboration with Pr. L. Strong and Dr. V. Huff (MD Anderson, Houston) who both showed a lot of interest in checking the status of nov in Wilms tumors. Screening of a HeLa cell cDNA library provided by Pr. P. Chambon had permitted C. Martinerie to clone the human nov ortholog which was used by V. Huff to probe a Northern blot of RNA species isolated from several diferent Wilm's tumors. The results obtained indicated that increased expression of nov was observed mainly in tumors showing a marked stromal aspect [21]. Most interestingly, the increased expression of nov was not always associated with Wilms tumor development but appeared to be dependent upon the origin and type of the tumors. The results obtained also suggested the existence of an inverse relationship between the expression of WT1 and nov [21].

This work led us to determine whether the transcription factor WT1 might regulate negatively the transcription of nov. With the help of G. Chevalier, a new PhD student in my laboratory, C. Martinerie who was given this project, cloned the nov promoter region and constructed several reporter plasmids. The results obtained indicated that WT1 downregulated indirectly the expression of nov [22]. The promoter region responsible for this effect did not contain consensus WT1 binding sites but contained several other sites for regulatory proteins that may potentially interact with WT1 and be responsible for the negative regulation of the nov promoter activity.

Because cyr61 and ctgf had been shown to be immediate early genes C. Martinerie and S. Middendorp, a master's student in our group, analysed the expression of nov upon stimulation of starved cells. Unfortunately, their results did not permit us to establish whether nov was an immediate early gene or not.

It is during these years that Dr. G. Scholtz in the laboratory of Pr. H. Hanafusa informed me that nov was among the genes whose expression was downregulated upon chicken embryo fibroblast transformation by the v-src oncogene. We met in New York to draw the lines of a collaboration that eventually led G. Scholtz to establish that the expression of nov was associated with cell quiescence and was downregulated upon serum stimulation of starved cells [23].

To me these results were extremely stimulating because they had important biological consequences: i) the association of nov expression with cell quiescence of CEF was in total agreement with the negative effects of nov on CEF proliferation that we had previously reported [13], ii) the nov gene was not an immediate early gene. The three proteins that had been proposed to constitute a new family of multimodular proteins (CCN) were showing distinct biological properties.

At this time, Dr. W. Zumkeller, a post doc in Pr. P. Schofield's laboratory, contacted me to undertake studies regarding the possible functional relationship between nov and IGFBPs. W. Zumkeller showed so much enthusiasm that I eventually got in touch with P. Schofield and offered to come to Cambridge and discuss this topic with both of them. The expertise of P. Schofield's in IGF biology and WAGR syndrome provided the basis for studying nov expression in Wilms tumors. G. Chevalier, was sent to P. Schofield laboratory in Cambridge to initiate the study of nov expression in Wilms tumors by in situ hybridization. The preliminary results that were obtained identified for the first time the blastemal cells as positive for nov expression in Wilms Tumors. These conclusions were not in full agreement with those reached previously with the Houston group.

### Ten years after MAV cloning: A new perspective

Dr. H. Yeger from the Hospital for Sick Children, Toronto who I met at the 1995 AACR meeting in Toronto, proposed to join our collaborative project and to perform immunohisto chemistry (IHC) on normal developing human embryo and on a wider panel of Wilms tumors. Because the Wilms tumors belong to a very heterogenous group of tumors, he suspected that the variability of the samples that were used in our different studies might account for the conflicted observations.

The proposed collaborative study was aimed to identify the nature of cells positive for nov and to establish whether the elevated expression of nov in tumors resulted from increased expression in tumor cells or from the clonal expansion of nov-expressing cells in the tumor. H. Yeger used the antibodies that we had raised against the C-terminal part of nov and the internal region of ctgf to establish the expression profile of these two proteins during human embryogenesis. Samples provided by P. Schofield permitted to identify the sites of nov and ctgf expression in normal first trimester human embryos whereas Wilms tumor samples from the Hospital for Sick Children were used to study nov expression in Wilms' tumors. G. Chevalier, was offered to join this project and was hosted by H. Yeger to complete this work and to confirm by IHC the preliminary in situ results that she had obtained with Dr. F. Dieterlin and Dr. L. Pardaneau in Nogent sur Marne. The work performed in collaboration with the group at Nogent had permitted identification of sites of nov expression in the chicken embryo from early stages to birth. The results that were obtained had identified the developing cartilage and the nervous system as major sites of nov expression in the chicken embryo whereas the urogenital system, muscle and vessel endothelium stained with less intensity.

The availabilty of a wide panel of tumor samples at The hospital for Sick Children permitted us to perform a comprehensive study of nov expression in Wilms tumors that shed new light on the role of nov in kidney tumorigenesis [24]. This study also established for the first time that the levels of nov RNA and protein were not always matching. Indeed, in spite of the low levels of nov RNA that were detected in kidney podocytes, high and increasing amounts of the nov protein accumulated in these cells during development. These results provided the first clue in favor of post transcriptional regulation of nov expression in normal tissues.

Based on the studies performed with the Wilms' tumors and with a series of rhabdomyosarcoma tumors and cell lines, H. Yeger had foreseen the implication that nov could be involved in the development of muscle. Confocal microscopy had shown co-localization of nov and desmin in a rhabdomyosarcoma cell line (RD) induced to undergo myotube differentiation in the presence of low serum [Chevalier, Yeger, Perbal; unpublished observations]. Interestingly, nov was associated with a high molecular weight complex in SDS-PAGE suggesting a very stable protein interaction. Induction of differentiation also produced an increase in the measurable level of nov. Based on these observations, H. Yeger, back in 1998, send us a detailed proposal in which he proposed a collaborative study aimed to better understand the role of nov in muscle differentiation. The completion of this project in my group was unfortunately hampered by later events (see below). It is now being pursued by previous members of my group who have left the laboratory.

Soon after his return to Germany, W. Zumkeller who had perceived the importance of this new developing fled, introduced me to one of his colleague, Pr. M. Westphal, at the Eppendorf clinic in Hamburg where I was presenting our recent results.

M. Westphal who had established several cell lines from human glioblastomas and astroctytomas showed a real interest in the expression profile of nov in the nervous sytem and he agreed to start a collaborative project whose first aim was to determine whether the levels of nov expression would be affected in his tumor cell lines, and whether nov might be a good marker for typing or prognosis of brain tumors. This project was given to Pr. Li Wen Xin who at that time, was staying as an invited Professor in my laboratory. The glioma cells showed a variable level of nov expression, with the lowest levels of nov being detected in the most agressive tumors. These results suggested for the first time, an inverse correlation between tumorigenicity and the expression of a ccn gene [25], an observation in agreement with the antiproliferative activty of nov on chicken embryo fibroblasts [13].

During one of my trips to China Pr. Li Wen Xin, organized a meeting in Chonqing with Pr. W. Cai who was interested in the nervous system development and was looking for genes differentially expressed in normal and pathological conditions. We both decided to undertake with Dr. Su an in situ analysis of ccn3 gene expression in the nervous system of human developing embryo. This study confirmed that nov expression was associated with the development of the nervous system in normal human embryos and permitted identification of the positive neurons in medulla horns the cerebral cortex and ganglia as major sites of nov expression [26]. At the same time, preliminary results were obtained suggesting that nov expression might be associated with the establishment of cognitive functions in normal rats [27]. The staining of nov in human nervous system was matching the results that we had previously obtained with the chicken model.

The unique human material provided by P. Schofield's collaborators permited H. Yeger to establish a comprehensive pattern for the nov and ctgf protein in human developing tissues and led to a thorough description of nov protein expression in normal human developing embryo [28].

The overlaping results that were obtained in chicken mouse and human and confirmed that nov expression was not restricted to the kidney and strongly suggested that nov was playing a role in differentiation.

For the sake of homogeneity that is required for a PhD defense, I suggested G. Chevalier to concentrate on the expression of ccn3 during the normal development of chicken kidney and in MAV-induced nephroblastomas. These studies were performed in close collaboration with C. Khelifi and V. Joliot who were in charge of identifying the viral and cellular determinants responsible for the high specificity of MAV tumorigenic potential. Studies performed by G. Chevalier and Pr. Y. Cherel in Nantes established that the blastemal cells which undergo epithelial differentiation are the target for MAV infection in the avian kidney (manuscript in preparation). The blastemal cells which were positive for ccn3 also express high levels of viral sequences and give rise to the tumor cells that develop later. Hence the concept emerged of MAV inducing the polyclonal proliferation of tumor cells that already express nov instead of increased nov expression being driven by enhancer sequences of the MAV LTR. To tackle this problem, and better understand the relationship that might exist between nov expression and the induction of nephroblastomas by MAV, we have undertaken an identification of MAV integration sites in the chicken genome at a larger scale. Because a series of pulse field electrophoresis performed by I. Joubert indicated that a limited number of MAV integration sites were represented in viral-induced nephroblastomas [10] we planned a strategy based on the use of bacterial artificial chromosomes (BACs) Unfortunately the initial attemps of I. Joubert to construct BAC libraries from tumor and normal genomic kidney DNAs failed. After discussing the question with Dr. R. Zoorob and Dr. C. Auffray at Villejuif, we agreed upon a collaborative approach based on the use of a BAC library of chicken DNA that had been constructed by R. Zoorob. As a first step in this approach, V. Joliot isolated a series of cellular DNA fragments which were contiguous to the MAV insertion sites previously identified in nephroblastomas.

While the work performed by G. Chevalier did not allow for unambiguous identification of the sites of nov expression in early stages of kidney development and in developing nephroblastomas, the studies performed by C. Khelifi eventually identified viral components involved in nephroblastoma development [29].

The screening of the BAC library that was pursued by C. L. li soon indicated that nov was not a common integration site for MAV in viral-induced nephroblastomas, a conclusion that was strengthened through physical mapping of MAV integration sites by fluorescent in situ hybridization (FISH) analysis [18]. Quite interestingly, a study performed on MAV2-induced tumors reached similar conclusions [30]. However, in most of the MAV2-induced tumors, the level of nov expression was not increased as compared to normal kidney. This recent result opened new questions regarding the biological significance of nov overexpression in avian tumors, and reinforced the conclusion drawn from the analysis of Wilms' tumors, that the nov acronym was not appropriate. Hence the need for a new nomenclature (see below).

Because the results obtained by G. Chevalier during her stay at Nogent sur Marne had pointed to cartilage and nervous system as major sites for ccn3 expression early during chicken development, I recruited Dr. M. Laurent, a senior scientist who was seeking a laboratory in which to work, and I offered her to study the role of ccn3 in the development of cartilage and nervous system differentiation, both in normal and pathological conditions. At the same time I hired a master's student, W. Barbot, to work with M. Laurent under my scientific supervision.

The role of nov in cartilage differentiation had been clearly documented by both G. Chevalier and W. Barbot. Their observations set the stage for further studies regarding the involvement of nov in the control of limbs growth, in concert with other members of the CCN family that had already been involved in chondrogenesis. Unfortunately, later events [31] interfered with the publication of these results which were included in a general review [3].

For many years, the multimodular organization of the CCN proteins and the oncogenic activation of nov resulting from its amino truncation had raised in my mind a considerable interest regarding the relationships existing between the structure and the functions of the CCN proteins.

To get a better insight into the biological properties of nov and the potential role of each individual module, two different approaches were undertaken. On one hand I had asked C. Martinerie to work out conditions for the purification of nov, and on the other hand, I used the two hybrid strategy to identify the proteins that physically interact with nov.

The procedure for purification of the CCN3 protein using Cibacron blue and reverse phase chromatography that was developed with Y. Bluquit at the Curie Institute, yielded low levels of protein in spite of using supernatants from SF9 insect cell cultures infected with a recombinant baculovirus driving the expression of CCN3. Switching to a 6-HIS tagged CCN3 protein appeared of potential interest to increase the purification yield. The construction of such recombinant clones was initiated later with C. Martinerie and E. Dulorme, an undergraduate student who spent some time to get trained in our team [32]

Because the insertion of MAV confered oncogenic properties on the amino truncated nov protein, we reasoned that the lack of the IGFBP-like domain present at the amino terminus of the CCN proteins might be responsible for the upregulation of cell growth. To check this hypothesis, we contacted Dr. M. Binoux and asked him to check whether CCN3 would bind IGF. The results obtained by ligand blotting were negative therefore suggesting that CCN proteins were not IGF binding proteins and were not involved in IGF signaling.

About a year later, the group of Dr. R. Rosenfeld re-discovered that CCN proteins were sharing partial identity with IGFBPs. Even though this relationship had been clearly mentioned and previously discussed in our publications and those from Pr. G. Grotendorst and Pr. L. Lau, an IGFBPr nomenclature was proposed for the CCN proteins. Following R. Rosenfeld's manuscript M. Binoux became interested in CCN proteins and introduced me to Pr. Y. Le Bouc who was supposed to take over his directionship. After I had presented our projects, Y. LeBouc who did not know much about the CCN proteins, realized the potential interest of the structural realtionship between IGFBPs and CCNs. We both agreed in 1998 that I would be a co-applicant on the renewal of the Inserm unit that was needed after M. Binoux retirement. Our two laboratories were showing complementary expertise; Le Bouc's was providing the IGF biology knowledge and access to patient samples, my group was bringing a solid molecular biology and the nov system. Furthermore, we had established with H. Yeger by immunofluorescence and western blotting of samples provided by P. Schofield that nov was highly expressed in adrenal. Le Bouc's group could provide human adrenal tumor samples that were needed to explore the potential deregulation of nov in these tumors. A new collaboration was born.

At that time, two colleagues, Pr. S. Kyurkchiev from Sofia and Pr. G. Thomopoulos from Thessaloniki joined my laboratory for a short time as invited Professors of the Paris 7 University.

Because the analysis of the nov protein in tumor samples required purified antibodies, I asked Pr Kyurkchiev who is a talented immunobiologist to join the adrenocortical tumor project. During his stay, Pr. Kyurkchiev purified the antibodies that were extensively used by C. Martinerie and M. Laurent to perform their analysis of the nov protein in normal and tumor tissues [31].

In the meantime, thanks to B. Roizman, I had spent a couple of month in his laboratory at the University of Chicago in 1997, to screen cDNA libraries of normal and tumor tissues to identify potential partners of nov. The continuous help and advice that I got from B. Roizman was extremely useful and permitted me to isolate several candidates, the partial sequencing of which identified integrins, calcium binding proteins, fibulin and a subunit of RNA polymerase II as interacting with nov. During this period, I decided to re-examine an observation that had puzzled me. Back in 1997, after I was invited to give a talk by Pr. Roy Burman at the USC Medical Center in Los Angeles, one of his colleagues who had evaluated our nov antibody claimed that it was not specific because it was staining the nucleus of HeLa cells. Even though this result was not in agreement with our conventional way of thinking about a secreted regulatory protein, I performed a series of immunostainings with two different sources of nov-antibody which indeed confirmed that the nucleus of two cancer cell lines (HeLa and 143 osteosarcoma cells) stained positive for nov. These observations eventually helped us to establish that a truncated nov protein was located in the the nucleus of these tumor cells whereas it was not detected in the VERO normal monkey kidney cells. Pr. B. Roizman suggested me to run confocal analysis on HSV infected cells with antibodies raised against ICP4 and ICP8, two viral proteins involved in transcription and replication, respectively. The colocalization of nov and ICP4 in HSV-infected cells strongly suggested that the nuclear nov variant might act as a transcriptional regulator.

The large number and the the great variety of potential nov partners were totally unexpected. Thanks to Dr. A. Sentenac, I could spend a few weeks in the Service de Biochimie et Genetique Moleculaire, at Saclay, where I could confirm the interaction of CCN3 with rpb7 of RNApolymeraseII, reisolate candidate clones and establish that the constitutive modules of nov were differentially implicated in these interactions. Most interestingly, the CT module was showing a high capacity to interact with some of the candidate partners. In 1999, I hired R. Sainson, a master degree student, to help me in this project. In a couple of month, thanks to D. Nolibe at the INSTN, we could confirm the interactions of nov with fibulin 1C and several other candidates.

After the move of my team to the Inserm, we could finalize this work with the help of C. Martinerie and establish that the C-terminal domain of nov was sufficient to permit its physical interaction with fibulin 1C [33]. These results confirmed our previous observations that the full length nov protein was localized in the extracellular matrix and provided the first clue for nov acting in cooperation with proteins involved in cell attachement and intercellular signaling. In this work we could also establish that an aminotruncated nov protein lacking the two first modules of CCN proteins was produced in the conditioned medium of insect cells transfected by a nov recombinant baculovirus. Inasmuch as the amino terminus of the truncated nov protein was identical to the aminoterminus of a previously described variant ctgf [34] we concluded that cleavage was resulting from a proteolytic cleavage involving a specific protease yet to be identified.

The two major perspectives open by this work came from the fact that i) the truncated nov protein was binding fibulin 1C with a much higher affinity than the full length nov protein, therefore suggesting that the ratio of truncated over full length forms might be critical in regulating the biological activities of nov, ii) the truncated form that was detected in the conditioned medium was structurally identical to the nuclear nov variant. Since no evidence for alternative splicing has been obtained as yet, this observation suggested that the truncated nov variant might be internalized by a mechanism yet to be identified [3].

With the help of Pr. G. Thomopoulos, a distinguished electron microscopist, we could confirm by the immunogold labeling that the nov protein could be detected both in the cytoplasm and in the nuclear pores of adrenocortical cells [35]. Furthermore, the nov protein detected in the cytoplasm was not embeded in vesicles, therefore reinforcing the potential existence of un conventional process responsible for internalization of the truncated protein generated by proteolytic digestion at the cell membrane [3].

### The beginning of a new era : the antiproliferative activity of CCN3 uncovered

It is during a trip in Toronto, on the way to meet Dr. H. Yeger, that I thought there was a strong need for an International Meeting about CCN genes and proteins. After discussing the matter with H. Yeger, who was very enthusiatic, I contacted G. Grotendorst and L. Lau. Both of them showed a great interest in this project. Back in France, I organized the First International Workshop on the CCN family of genes that was to be held from October 17–19, 2000 in Saint Malo. This bi-annual meeting became a must have event because it was fulfilling the needs of many colleagues in the field to discuss and present their new results in a friendly atmosphere.

During the preparation of this meeting, G. Grotendorst, L. Lau and myself agreed to write a scientifically based rebuttal to the proposal of Rosenfeld to rename CCN proteins [36,37]. Recent results have now confirmed that indeed, the IGFBP domain of nov does not function as an IGF binding domain in the context of IGFBP3 [38].

The proposal for a unique CCN nomenclature was discussed and approved at the first workshop [39]. The CCN nomenclature found its justification in the variety of names attributed to the same CCN proteins, and the confusion that resulted from names which were often misleading, restrictive, or inappropriate [40]. The International CCN society [41] was created to consolidate and favor relationship and scientific exchange between worldwide members. In my opinion, these two events were pivotal for the recognition of the CCN field as a whole.

CCN3 was born.

B. Cadot, a new master student in my laboratory got involved in two collaborative studies in which we examined the potential usefulness of CCN3 detection in human prostate tumors and renal cell carcinoma (RCC) for prognosis, typing and therapy.

The results that were obtained in collaboration with Dr. R. Tatoud at Norwich established that a higher expression of CCN3 was associated with more advanced stages of prostate tumors, and suggested that in this system, increased proliferation that occurs upon transformation of prostate cells correlates with CCN3 expression [42]. Similarly, the analysis of CCN3 expression that was performed with Dr. AF Gogel, in a series of cell lines representing increasing grades of RCC, led to the conclusion that in these cells an elevated expression of CCN3 was associated with higher grades and better ability to develop tumors in SCID mice [43].

While I was hosted by Pr. B. Roizman and Pr. R. Weichselbaum for another short stay at the University of Chicago, we examined in greater detail preliminary conflicting data that had been obtained by M. Laurent in my own laboratory and by H. Wang in R. Weichselbaum's. A few stable transfectants had been isolated from the G59 glioma cell line by M. Laurent when she was working in my group. While M. Laurent claimed that the expression of ccn3 did not have any obvious phenotypic effects, Dr. Wang observed that expression of ccn3 in these same cells altered their proliferation and tumorigenic potential. Experiments were repeated during my stay and the results that were obtained clearly established that the expression of ccn3 reduced significantly both cell proliferation and tumorigenicity [44].

To me, these results were of prime importance because i) they confirmed the previous observations of Dr. Li which established an inverse relationship between tumor agressiveness and expression of ccn3 [25], and ii) they suggested that the biological effects of ccn3 might be dependent upon the cellular context since in prostate carcinoma and RCC cells the relationship was direct.

At that time, Dr. A. Lombet and V. Martinez, a PhD student in my laboratory got the first evidence that the addition of recombinant CCN3 protein to SKNSH cells in culture resulted in a marked increase of intracellular concentration of calcium ions. Since C.L. Li had confirmed that CCN3 was interacting with the calcium binding protein S100A4, the effects of CCN3 on calcium concentration had a lot of potential meanings [45,46]. Upon the suggestion of A. Lombet, we then investigated whether CCN3 had an effect on ion channeling by performing patch clamp with the help of Pr. D. Tritsch and P. Vincent in Paris. Not only did the results that we obtained confirm that the increased calcium uptake was accompanied by a burst of voltage-dependent K+ (BK) current, but they also established that CCN3 was blocking sodium channels very efficiently [47].

Since the addition of CCN3 triggered calcium uptake by a non-voltage dependent channel and/or ER calcium mobilisation in a cell specific way [45] it would be interesting to check whether the different effects that were observed with the various cell types that we used were in any way related to the quite different levels of endogenous ccn3 expressed by these cells.

In nany case, these results conferred for the first time a biological activity on CCN3 and established CCN3 as a genuine signaling protein.

Because the elevated expression of CCN3 in transformed cells appeared to vary with the origin of the tumors, it was quite interesting to establish the pattern of CCN3 expression in a much wider range of tumors. For this purpose, I engaged my laboratory in a series of collaborations that proved extremely fruitful.

### Twenty years after MAV cloning: the picture is getting in focus

From the immunocytochemistry analysis and RNA profiling that was performed on various tumor types we could draw the following conclusions. An elevated expression of CCN3 was associated with tumor differentiation and good prognosis in the case of Wilm's tumors, chondrosarcomas, osteosarcomas, neuroblastomas and chronic myeloid leukemia, whereas it was associated with an increased proliferation rate and/or metastases in the case of RCC, prostate carcinomas, and Ewings tumors [24,39,43,48-50].

The study performed with B. Alman's group also established for the first time that the expression of CCN3 was indeed required for cartilage differentiation [49].

It was also shown in two other collaborative studies using either constitutive or inducible expression vectors, that the ectopic production of ccn3 induced a dramatic reduction of cell proliferation in all the tumor cells tested [51,52]. Therefore, the antiproliferative activity of CCN3 was not restricted to glioma cells but could be considered as a genuine common feature. Along this line, it will be interesting to determine whether the effects of CCN3 on intracellular calcium concentration are responsible for its growth inhibitory effects.

The case of Ewing tumors was of particular interest because in this sytem CCN3 was acting as a double edge sword. On one hand, the expression of ccn3 reduced cell proliferation, but on the other hand, the expression of ccn3 in primary tumors was associated with an increased risk of developing metastases [48,52].

The identification of new partners for CCN3 provided important clues regarding its mode of action.

Among the many potential partners for ccn3 that I had isolated in the two hybrid screen performed in B. Roizman's laboratory, were integrins, notch, a ligand of Zo1 protein, and a few nuclear proteins.

It is during a trip in Tokyo, where I was invited to give a talk by Pr. J. Ikawa, that I met Dr. K. Katsube who expressed a strong interest for the ccn3 gene expression pattern and its possible relationship with the Notch receptor. A very fruitful collaboration was set up and a few years later, Notch1 was shown to physically interact with CCN3 [53-55].

During the same period of time, I was contacted by A. Gelhaus in Essen, who discovered that choriocarcinoma cells induced to express connexin43 were upregulating the expression of ccn3. From the collaboration that I had initiated with C. Naus along the study of gliomas [44] I realized that there might be a tight link between the expression of ccn3 and connexins and I suggested A. Gelhaus to check whether the forced expression of ccn3 would have any effect on connexin 43. At the same time, C. Naus suggested that connexin 43 and CCN3 might interact. In two simultaneous publications, we reported that the physical interaction of CCN3 with connexin and inter-connected expression of these two genes, might provide important clues for understanding the role of connexins in cancer. Indeed, cells lacking Cx43 expressed very low levels of CCN3, grew faster and were more tumorigenic than the cells which were positive for Cx43 and expressed CCN3 [51,56].

From these studies, it appeared that CCN3 was physically interacting with many different proteins, as suggested by the initial results of the two hybrid screening. The different nature and subcellular localisation of these partners was probably the reason why CCN3, as other CCN proteins, was involved in such a variety of biological functions [57]. Because CCN3 was obviously involved at different levels of cell signalling, I proposed that CCN3 might be engaged in multimolecular complexes, where it could play a scaffolding-type of function, allowing the coordination of different signaling pathways [3,58]. In this model, the functions and sites of actions of CCN3 are determined by combinatorial events that depend upon the bioavailability of the various components involved in constitution of the complexes. Multifunctional complexes have already been reported to play critical roles in the biology of eucaryotic cells.

Another fundamental aspect of the CCN3 biology emerged from our recent studies.

From the early report of CCN3 detection in nuclear extracts and interaction of CCN3 with RNA polymerase II subunit 7 [59], we have explored the possible functions of aminotruncated CCN3 variants in the regulation of transcription.

Work performed by N. Planque, an Associate Professor in my laboratory, recently established that any alteration of the aminoterminus of the CCN3 protein that would result in the loss of the signal peptide would also result in the nuclear addressing of the variant CCN3 [60]. This situation is not unique to CCN3 since a number of proteins that are involved in outside signaling have been detected in the nucleus of eucaryotic cells. Although the biological significance of this dual localisation is not always clear, it is opening up very interesting perspectives in cell biology. In the case of CCN3, N. Planque established that the CT domain contains a nuclear localisation signal that can drive the aminotruncated CCN3 proteins to the nucleus. Furthermore, the CT domain of CCN3 confers on the protein the capacity to inhibit transcription [60].

These results are of considerable importance in the light of the transforming activity of the truncated CCN3 protein that was expressed in MAV-induced nephroblastoma [13]. Truncation of the CCN3 protein might occur under chromosomal rearrangements and mutagenic events that occur during tumor progression. Alternatively, the well-documented increased production of proteases by tumor cells might also lead to large quantities of the truncated CCN3 variant that are detected in the conditioned medium of CCN3-expressing cells [61] and consecutive increased internalization of these variants. Along this line, we have proposed that a balanced production of full length growth-inhibitory CCN3 protein and of truncated growth-stimulatory CCN3 truncated variant is required for appropriate cell proliferation and differentiation. Any event disrupting this balance would result into abnormal signaling that might participate in the establishment or maintenance of the tumor state. Whether this situation also applies to other CCN proteins is a challenging question.

### The near future ..

At a first glance it may seem that the variety of functions, interactions, and sites of expression that have been assigned to CCN3 make it extremely difficult to categorize it. The pleiotropic functions of CCN3 likely reflect its ability to interact with several key regulatory proteins and ligands and its central place in the control of cell signaling.

As yet the antiproliferative activity of CCN3 is the only function that is common to all situations in which the biological activity of CCN3 has been assessed. The recent demonstration that the antiproliferative activity of CCN3 constitutes a critical factor in the control of 3D spatial localisation of melanocytes [62], provides the first example of a biological situation in which this inhibitory effect is required for the maintenance of a normal phenotype in human skin. Work which is in progress with other normal tissues, is expected to provide more examples that will confirm the central role of CCN3 in the control of normal cell behavior.

Studies performed on tumor cells have allowed to pinpoint several aspects of CCN3 biology that are altered upon initiation or during progression of cancers. Future challenges will include the use our partial knowledge in the development of new tools for molecular diagnosis and therapy.

Preliminary results that have been obtained in various pathological situations aside from cancer, have also pointed CCN3 as a key regulator of potential interest in molecular medicine [47]. The production of a recombinant protein of reliable quality should permit us to proceed along the way of targeted therapy. This aspect will require energy, talent and inventiveness.

The discovery of CCN3 as an antiproliferative protein involved in the control of normal growth offers enormous potential interest for translational research. I strongly believe that in the near future, the CCN family of proteins will get much more attention. In my opinion, the assignment of a universal essential function such as the growth inhibitory effect of CCN3, might help considerably to attract new scientists in this relatively new field.

## Conclusion

In this historical review, I have presented a personal accounting of what I consider as a very exciting and challenging scientific journey. It is not meant  to be a totally encompassing scientific accounting of the CCN3 field, as we all  do appreciate the significant participation of others. Although the focus of this review was on CCN3, it is quite obvious to me that it is necessary to consider its biological properties in the context of the whole family of CCN proteins [63]. One can easily predict that physical and functional interactions between the different members of the CCN family of proteins should permit fine tuning of their individual functions both in normal and pathological conditions.

It may seem a long road to go but I am quite confident that the growing interest in CCN proteins will fuel our progress at an, as yet, unexpected pace and efficiency.
